# A genetic interaction map centered on cohesin reveals auxiliary factors involved in sister chromatid cohesion in *S. cerevisiae*

**DOI:** 10.1242/jcs.237628

**Published:** 2020-05-22

**Authors:** Su Ming Sun, Amandine Batté, Mireille Elmer, Sophie C. van der Horst, Tibor van Welsem, Gordon Bean, Trey Ideker, Fred van Leeuwen, Haico van Attikum

**Affiliations:** 1 Department of Human Genetics, Leiden University Medical Center, Einthovenweg 20, 2333 ZC, Leiden, Netherlands; 2 Electrical Engineering, Mathematics and Computer Science, Delft University of Technology, 2600 AA, Delft, Netherlands; 3 Division of Gene Regulation, Netherlands Cancer Institute, Plesmanlaan 121, 1066 CX, Amsterdam, Netherlands; 4 Bioinformatics and Systems Biology Program, University of California, San Diego; La Jolla, CA, 92093, USA; 5 Department of Medicine, Division of Genetics, University of California, San Diego; La Jolla, CA, 92093, USA; 6 Department of Bioengineering, University of California, San Diego; La Jolla, CA, 92093, USA; 7 Cancer Cell Map Initiative (CCMI), Moores UCSD Cancer Center, La Jolla, CA, 92093, USA

**Keywords:** Genetic interaction mapping, Cohesin, Sister chromatid cohesion, Prefoldin, Irc15, Cohesinopathy

## Abstract

Eukaryotic chromosomes are replicated in interphase and the two newly duplicated sister chromatids are held together by the cohesin complex and several cohesin auxiliary factors. Sister chromatid cohesion is essential for accurate chromosome segregation during mitosis, yet has also been implicated in other processes, including DNA damage repair, transcription and DNA replication. To assess how cohesin and associated factors functionally interconnect and coordinate with other cellular processes, we systematically mapped the genetic interactions of 17 cohesin genes centered on quantitative growth measurements of >52,000 gene pairs in the budding yeast *Saccharomyces cerevisiae*. Integration of synthetic genetic interactions unveiled a cohesin functional map that constitutes 373 genetic interactions, revealing novel functional connections with post-replication repair, microtubule organization and protein folding. Accordingly, we show that the microtubule-associated protein Irc15 and the prefoldin complex members Gim3, Gim4 and Yke2 are new factors involved in sister chromatid cohesion. Our genetic interaction map thus provides a unique resource for further identification and functional interrogation of cohesin proteins. Since mutations in cohesin proteins have been associated with cohesinopathies and cancer, it may also help in identifying cohesin interactions relevant in disease etiology.

## Abstract (precis)

**Highlighted Article:** The characterization of auxiliary factors in sister chromatid cohesion as identified by genetic interaction mapping in yeast, reveals novel roles for the prefoldin complex and the microtubule-associated protein Irc15.

## INTRODUCTION

Sister chromatid cohesion ensures close proximity of the two sister chromatids from the time of replication until their separation to opposite spindle poles during mitosis. Sister chromatid cohesion is mediated in all eukaryotic cells by a multiprotein complex called cohesin (Michaelis et al., 1997). In budding yeast (*Saccharomyces cerevisiae*), Smc1, Smc3, Scc1 and Scc3 make up the core of the cohesin complex, which is loaded onto chromatin during G1 phase. It forms a ring-like structure that encircles sister chromatids generated during DNA replication in S phase in a manner dependent on Smc3 acetylation by Eco1. Subsequently the cohesive status is sustained throughout G2 and M phase by several maintenance factors, including Rad61, Pds5 and Sgo1. Several accessory proteins have also been implicated in promoting sister chromatid cohesion, including Elg1, Ctf18, the alternative replication factor C (RFC) complexes, the replisome component Ctf4, the Chl1 helicase-like protein, the chromatin remodeler Chd1 and the S phase checkpoint proteins Mrc1 and Tof1 (Petronczki et al., 2004; Parnas et al., 2009; Hanna et al., 2001; Skibbens, 2004; Xu et al., 2004; Boginya et al., 2019). Finally, sister chromatid cohesion is dissolved at the metaphase to anaphase transition by proteolytic activity of Esp1 towards Scc1 (Uhlmann et al., 1999; Cohen-Fix et al., 1996; Xiong and Gerton, 2010).

Besides ensuring proper chromosome segregation, cohesin has been shown to impact the repair of DNA double-strand breaks (DSBs) (Unal et al., 2004, 2007; Strom et al., 2004; Heidinger-Pauli et al., 2009; Gelot et al., 2016; Wu et al., 2012; Kong et al., 2014), gene expression (Gullerova and Proudfoot, 2008; Dorsett, 2011; Lengronne et al., 2004) and nuclear organization (Harris et al., 2014; Yamin et al., 2020). In addition, several developmental disorders have been causally linked to germline mutations in cohesin genes and are collectively referred to as cohesinopathies. These include Cornelia de Lange syndrome (Deardorff et al., 2012; Liu and Baynam, 2010), Roberts syndrome (Vega et al., 2005) and Warsaw breakage syndrome (van der Lelij et al., 2010). Somatic mutations in cohesin genes, on the other hand, have been found with high frequency in various types of cancer (Thol et al., 2014; Bailey et al., 2014; Repo et al., 2016; Deb et al., 2014), underscoring the importance of cohesin genes in the development of pathogenesis. However, despite the important role that cohesin genes play in various cellular processes, including those relevant to disease manifestation, our understanding of how the cohesin complex functionally interconnects with these processes is still rather limited.

Genetic interaction screens have highlighted the connectivity between genes and their corresponding pathways, thus providing insight into the biological role(s) of individual genes (Mani et al., 2008). In yeast, such screens have led to the identification of new genes that contribute to efficient sister chromatid cohesion (Mayer et al., 2004; Chen et al., 2012), and provided valuable insight into the connectivity between cohesin genes and genes involved in DNA repair and DNA replication (McLellan et al., 2012; Warren et al., 2004). However, these studies were focused on a rather limited number of cohesin genes. Here, we examined genetic interactions between 17 different cohesin genes and more than 1400 genes involved in various biological processes in a quantitative manner. The resulting genetic interaction map describes novel connections for cohesin genes in various cellular processes, including post-replication repair, microtubule organization and protein folding, and reveals that the microtubule-associated protein Irc15 and prefoldin complex members Gim3, Gim4 and Yke2 are novel regulators of sister chromatid cohesion. Thus, we provide a unique and powerful resource for the identification and functional interrogation of cohesin proteins.

## RESULTS

### Mapping genetic interactions of cohesin

**Fig. 1.**
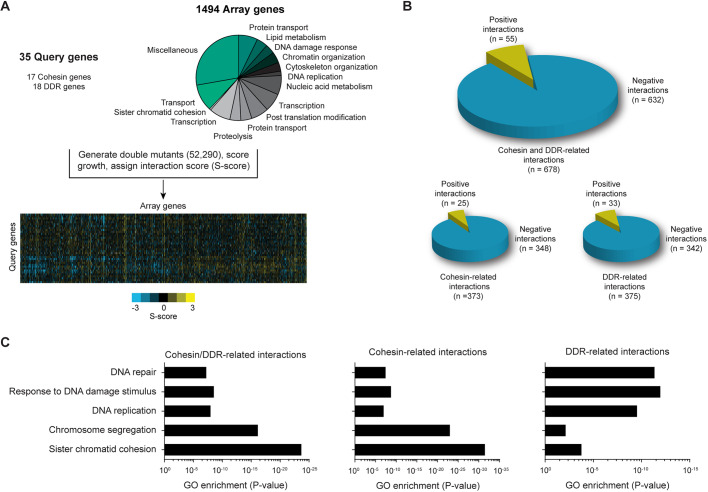
**A genetic interaction map centered on cohesin.** (A) Outline of the genetic interaction screen. Mutants in 17 cohesin and 18 DNA damage response (DDR) query genes were crossed against a panel of 1494 mutants in array genes involved in various biological processes. Genetic interactions were scored by quantification of colony sizes, providing each double mutant with a quantitative S-score. (B) Total number of positive (S-score ≥2) and negative (S-score ≤−2.5) interactions for all query (top), cohesin (bottom left) or DDR (bottom right) genes. (C) GO term enrichment of interactions involving all (left), cohesin (middle) or DDR genes (right).

To gain more insight into the relationship between sister chromatid cohesion and other cellular processes, a comprehensive genetic interaction map centered on cohesin was generated. To this end, query strains carrying gene deletion or temperature-sensitive alleles of 17 different cohesin genes and 18 DNA damage response (DDR) genes (Table S1) were crossed by using the synthetic genetic array (SGA) methodology (Tong and Boone, 2006) against a panel of 1494 array strains (Table S2) carrying gene deletion or decreased abundance of mRNA perturbation (DAmP) alleles of genes that represent various biological processes (Fig. 1A). We previously used the 18 DDR mutants to map interactions of the DDR network, and included these in the current study to warrant quality control and quality assurance (Guenole et al., 2013; Srivas et al., 2013). Genetic interactions were scored by quantifying colony sizes of the double mutants, which were normalized and statistically analyzed to provide each mutant with a quantitative S-score (Fig. 1A). S-scores ≤−2.5 represent negative or synthetic sick/lethal interactions, whereas S-scores ≥2 represent positive or alleviating/repressive interactions (Costanzo et al., 2019; St Onge et al., 2007; Hartman et al., 2001). In total, the profile map contains S-scores for 52,290 gene pairs (Fig. 1A; Table S3). Several routine quality control metrics were employed to ensure a high-quality map (Fig. S1). We observed a correlation of at least 50% between the genetic interactions identified in our screen and previously published genetic interaction maps (Fig. S1A,B) (Guenole et al., 2013; Collins et al., 2010; Costanzo et al., 2010). In addition, genetic interactions with the highest S-scores showed a high enrichment of interactions present in the Biogrid database (Fig. S1C).

Our genetic interaction map revealed in total 678 interactions, including 55 positive and 632 negative interactions (Fig. 1B). Validation of ∼70 interactions resulted in an overall false discovery rate (FDR) of 31% (Fig. S1D–G). In particular, we identified 348 negative and 25 positive interactions for the cohesin-related genes along with 342 negative and 33 positive interactions for the DDR genes (Fig. 1B). As expected, interactions found in the cohesin-associated group were highly enriched for the Gene Ontology (GO) terms ‘sister chromatid cohesion’ and ‘chromosome segregation’, whereas interactions for the DDR-associated genes were enriched for DNA repair-related GO terms (Fig. 1C; Tables S4–S6). In conclusion, a high-quality genetic interaction map centered on cohesin was generated, providing a useful resource to mine for crosstalk between sister chromatid cohesion and other cellular processes.

### Cohesin genes interconnect with genes involved in various biological processes

**Fig. 2.**
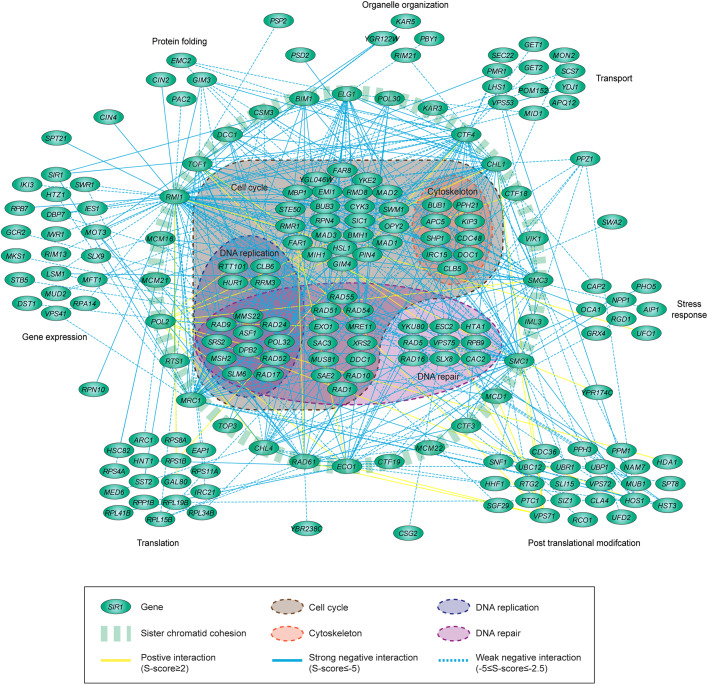
**A genetic interaction network centered on cohesin.** Visualization of significant genetic interactions of cohesin-related genes. Interacting genes were grouped based on GO annotation.

To better understand the complexity of the interplay between sister chromatid cohesion and other biological processes, we generated a genetic interaction network comprising interactions with S-scores ≤−2.5 and ≥2 for the cohesin-related query genes (Fig. 2). This interaction network may be relevant for other species as the vast majority of genes are orthologous to both fission yeast and human genes (Table S7).

As expected, we observed a strong relationship between sister chromatid cohesion factors and genes involved in cell cycle control (e.g. *SIC1*, *CTF19*, *BUB1* and *BUB3*), as well as in DNA replication (e.g. *RTT101*, *MMS22* and *POL2*), which is in agreement with the required coordination of these three processes to guarantee faithful chromosome duplication and segregation (Lengronne and Schwob, 2002; Fernius and Marston, 2009; Alexandru et al., 1999; Zhang et al., 2017; Edwards et al., 2003). Our network also revealed several known interactions between cohesin factors, mainly the non-essential cohesin accessory factors, such as *ELG1*, *TOF1* and *RMI1*, and genes involved in DSB repair (e.g. *RAD51*, *RAD52* and *SRS2*) (Ben-Aroya et al., 2003; Chang et al., 2005; Kanellis et al., 2003). Moreover, several interactions between cohesin factors and chromatin remodeling or histone-modifying complexes, such as *ASF1*, *IES1*, *HTZ1*, *SWR1*, *HDA1* and *HST3*, strengthen the link between sister chromatin cohesion and chromatin architecture (Huang et al., 2004; Huang and Laurent, 2004; Munoz et al., 2019; Sharma et al., 2013; Thaminy et al., 2007). Finally, we found a strong interplay between both essential and non-essential cohesin genes and genes encoding ribosomal subunits such as *RPL15B*, *RPBL41B* and *RPBL19B*. This is consistent with recent findings showing that defects in cohesin genes lead to defects in the production of ribosomal RNA and translation efficacy in both budding yeast and patient cells (Sun et al., 2015; Bose et al., 2012; Xu et al., 2014; Lu et al., 2014).

Our network also revealed several unanticipated interactions (Fig. 2). For example, several interactions between cohesin factors and genes involved in nucleotide excision repair, such as *RAD16* and *RAD1* with *SMC1* and *RAD10* with *RAD61*, in mismatch repair, such as *MSH2* with *MDC1* and *RAD61*, or in template switching, such as *RAD5* with *DCC1* and *RMI1*, might indicate a novel role for cohesin in post-replication repair. Supporting this notion, the separase complex is required for cohesin dissociation during post-replicative DNA repair (Nagao et al., 2004; McAleenan et al., 2013). Moreover, Smc1 is phosphorylated in an ATR-dependent manner after exposure to ultraviolet (UV)-induced DNA damage and the *smc1-259* mutant shows a high sensitivity to UV (Garg et al., 2004; Kim et al., 2002). Finally, several other unanticipated interactions were found between cohesin factors and genes involved in microtubule organization and protein folding, highlighting potential novel functional connections. Taken together, our genetic interaction map provides a resource of known as well as novel interactions between cohesin and genes involved in various biological processes, which may serve as a starting point for unraveling cohesin functions in these processes.

### Irc15 promotes the loading of centromeric cohesin

**Fig. 3.**
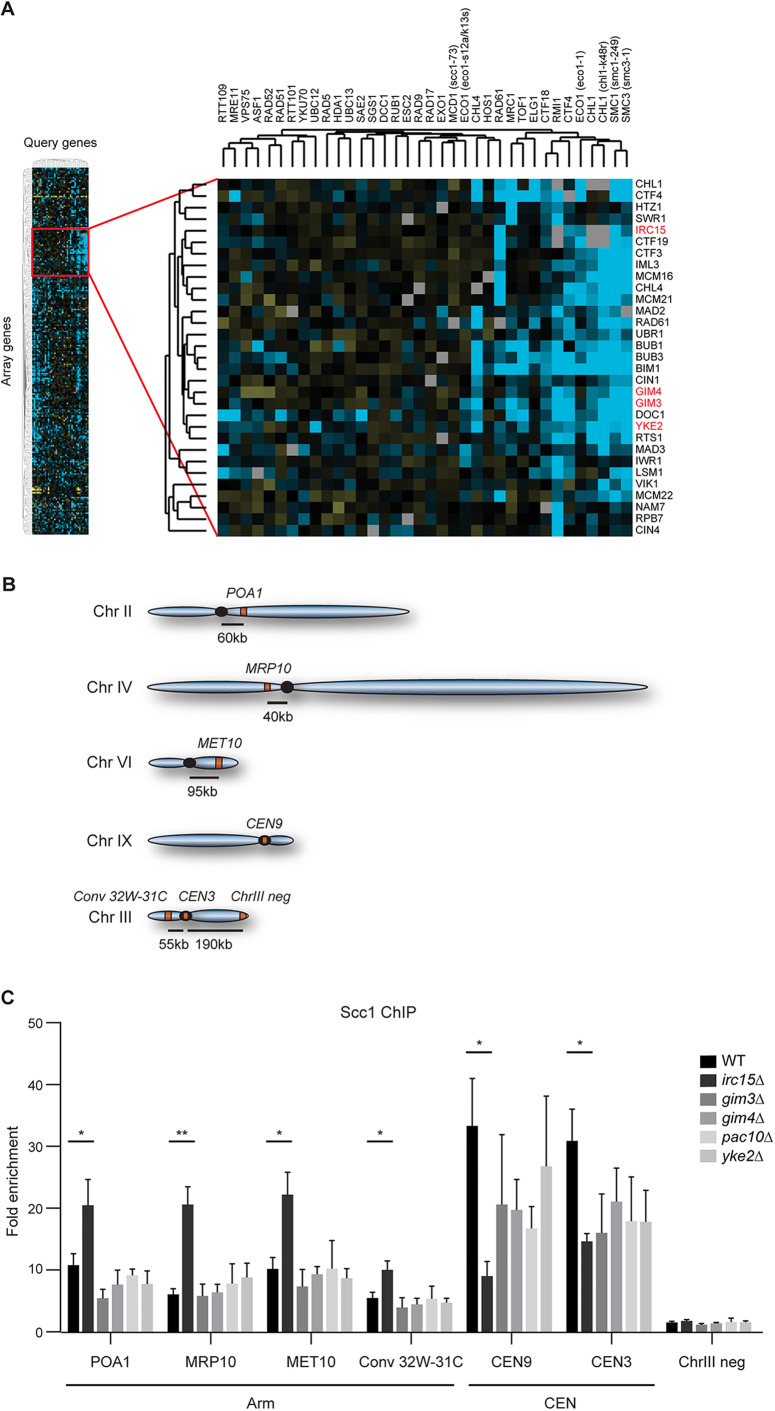
**Identification of new cohesin factors with Irc15 as cohesin loader.** (A) Heatmap displaying hierarchical clustering of genetic interactions scores (S-scores; left panel) identified a cluster of negative interactions involving cohesin factors and genes involved in chromosome segregation (right panel; blue, negative interaction; yellow, positive interaction; black, neutral interaction; gray, missing interaction). Potential new sister chromatid cohesion factors are highlighted in red. (B) Schematic of chromosomal loci assayed for Scc1 loading. qPCR was performed at known cohesin binding sites either on centromeres (*CEN9* and *CEN3*) or genic (*POA1*, *MRP10* and *MET10*) and intergenic (*Conv 32W-31C*) regions on chromosome arms. *ChrIII neg* was a negative control. (C) Enrichment of Scc1–Myc assessed by ChIP-qPCR at the indicated loci in nocodazole-arrested strains. Enrichment corresponds to the ratio of the Scc1–Myc signal over that found with beads alone. Mean±s.e.m. enrichment for three (*gim3Δ*, *gim4Δ*, *yke2Δ* and *pac10Δ*) or four (WT, *irc15Δ*) independent experiments is shown. *P<0.05; **P<0.01 (Student's *t*-test).

The cohesin interaction network may not only reveal new connections between cohesin genes and distinct biological processes, but may also uncover new factors involved in sister chromatid cohesion. Since genes acting in the same pathway tend to have similar genetic interaction profiles, we employed unsupervised hierarchical clustering of genetic interactions involving both cohesin and DDR-related query genes (Fig. 3A, left panel). Strikingly, a cluster of array genes interacted specifically with the cohesin query genes, which clustered separately from the DDR query genes (Fig. 3A, right panel). Interestingly, within this cluster, genes implicated in the establishment of pericentromeric cohesion, namely *CTF19*, *IML3* and *CHL4*, clustered together but did not interact with the three non-essential cohesin factors *MRC1*, *TOF1* and *ELG1.* While this cluster furthermore included genes implicated in chromosome segregation (e.g. *BIM1*, *MAD2* and *BUB1*), it was mostly dominated by genes involved in sister chromatid cohesion. Interestingly, among the genes in this cluster were also four genes, *GIM4*, *GIM3* and *YKE2*, that were all members of the prefoldin complex, and *IRC15*, a microtubule-binding protein, whose role in this process was unknown. We confirmed the negative genetic interactions of *gim3Δ*, *yke2Δ* and *irc15Δ* with *smc3-1*, and of *gim4Δ* and *yke2Δ* with *smc1-249* at semi-permissive temperature (Fig. S2). To assess their role in sister chromatid cohesion, we first examined whether *GIM4*, *GIM3*, *YKE2* and *IRC15* affect the loading of cohesin onto chromosomes. *PAC10*, which encodes another member of the prefoldin complex, did not display any significant negative interaction with cohesin genes and was therefore included as a negative control. Scc1 loading was assessed by chromatin immunoprecipitation (ChIP) at known cohesin-binding sites in G2 cells (Fig. 3B,C). A region on chromosome III devoid of Scc1 was used as a negative control (Pal et al., 2018). Scc1 loading was comparable in wild-type (WT) cells and cells lacking *GIM3*, *GIM4*, *YKE2* or *PAC10*, suggesting that the prefoldin complex is not involved in cohesin loading. However, Scc1 levels were decreased at centromeric regions in the absence of *IRC15*, while they were increased on chromosome arms, indicating that Irc15 regulates the distribution of cohesin on chromosomes. The defect in centromeric cohesin loading in *irc15Δ* may stem from a translocation of cohesin from the centromeres to the chromosome arms. However, we could not detect any such translocation of Scc1 by ChIP when cells proceeded from G1 phase to G2/M phase (Fig. S3A–F). Thus, we identify Irc15 as a new factor involved in the loading of centromeric cohesin. Interestingly, *irc15Δ* cells present a delayed pre-anaphase mitotic entry due to defective kinetochore–microtubule attachments (Keyes and Burke, 2009). Potentially, reduced cohesin loading and, consequently, impaired sister chromatid cohesion may have affected the maintenance of kinetochore–microtubule attachments during mitosis. To address this, we examined whether overexpression of Scc1 could rescue the kinetochore assembly defects observed in the absence of *IRC15* (Keyes and Burke, 2009). To this end, we monitored binding of the kinetochore-associated Ndc80 complex, which is involved in kinetochore assembly (McCleland et al., 2003), by performing ChIP of GFP-tagged Ndc80 at four different centromeres (CEN2, CEN3, CEN4 and CEN8) and a negative control locus (Neg1p2) (Lefrancois et al., 2013) in WT and *irc15Δ* strains carrying a galactose-inducible allele of *SCC1* (Fig. S3G). We found that Ndc80 binding was increased ∼4-fold in the absence of *IRC15* (Fig. S3H), indicative of a kinetochore assembly problem and agreeing with a previous observation (Keyes and Burke, 2009). Importantly, Ndc80 binding was not affected by Scc1 overexpression (Fig. S3H), suggesting that reduced cohesin loading in the absence of *IRC15* may not affect the maintenance of kinetochore–microtubule attachments.

### The prefoldin complex is involved in sister chromatid cohesion

**Fig. 4.**
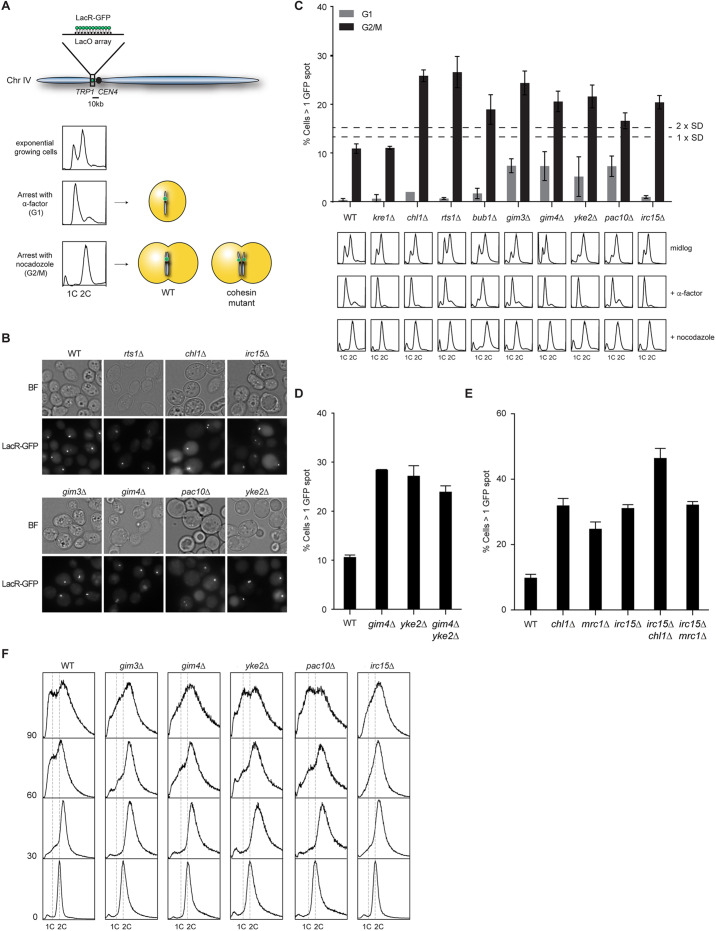
**The prefoldin complex and Irc15 affect cohesion establishment.** (A) Schematic of the sister chromatid cohesion assay. A LacO array was integrated on chromosome IV 10 kb away from *CEN4* in cells expressing the LacR–GFP fusion protein. Upon synchronization of the cells in G1 with α-factor or in G2/M with nocodazole, cells with normal sister chromatid cohesion show one spot in G1 and G2/M in the majority of the cells. Cohesin mutants show a larger fraction of cells with two GFP spots. (B) Representative images of the sister chromatid cohesion assay in nocodazole-arrested cells. (C) Quantification of sister chromatid cohesion in cells from B. The mean±s.e.m. percentage of cells with more than one GFP spot (top) is shown; ∼400 cells were scored in at least three independent experiments for each strain. Flow cytometry analysis of DNA content was used to monitor cell synchronization (bottom). (D,E) Quantification of sister chromatid cohesion in the indicated cells as in B. (F) Flow cytometry analysis of M phase progression of the indicated strains. Cells were arrested in G2/M by nocodazole treatment, released in YPAD and analyzed at the indicated timepoints.

While Irc15 promotes the loading of centromeric cohesin, its contribution to sister chromatid cohesion is unclear. Also unclear is whether the prefoldin complex affects this process. To examine this, we employed a strain in which a tandem LacO array was integrated 10 kb away from the *CEN4* locus and a LacR–GFP protein, which binds to the LacO array, is stably expressed (Fig. 4A). An increased number of G2/M cells with more than one GFP focus indicates a defect in sister chromatid cohesion in this strain (Fig. 4A,B). In our assays, a *kre1Δ* mutant defective in β-glucan assembly was included as a negative control, while *chl1Δ*, *bub1Δ* and *rts1Δ* mutants served as positive controls (Kitajima et al., 2005, 2006). As expected, two GFP foci were evident in ∼10% of the *kre1Δ* cells in G2/M phase, which was comparable to that in WT cells (Fig. 4C, top). In contrast, at least ∼20% of the *chl1Δ*, *bub1Δ* and *rts1Δ* cells displayed two GPF foci, indicative of a cohesion defect. Importantly, at least 20% of the *gim3Δ*, *gim4Δ*, *yke2Δ*, *pac10Δ* and *irc15Δ* cells showed more than two GFP foci, suggesting a defect in sister chromatid cohesion. It is noteworthy that an increased number of the prefoldin mutant cells also harbored two GFP spots in G1 phase. This may result from chromosome mis-segregation during the previous mitosis, which might be a consequence of defective cohesion (Hoque and Ishikawa, 2002; Sonoda et al., 2001), although we could not detect any aneuploidy in these mutants (Fig. 4C, bottom), likely due to the low frequency of these events (<10%). To determine whether the prefoldin holocomplex is involved in cohesion establishment, we compared sister chromatid cohesion in *gim4Δ* and *yke2Δ* single and double mutants (Fig. 4D). *gim4Δ* and *yke2Δ* were epistatic with regard to their cohesion defect, suggesting that the prefoldin complex as a whole functions in the same pathway for cohesion establishment. In addition, we also evaluated whether Irc15 functions in one of the two parallel non-essential cohesion pathways or defines a new cohesion pathway (Xu et al., 2007). To this end, we generated double mutants of *IRC15* with *CHL1* or *MRC1*, which encode components of the cohesion pathways involving Csm3 and Ctf18–RFC, respectively (Xu et al., 2007). While *irc15Δ* was epistatic with *mrc1Δ*, it displayed additive cohesion defects with *chl1Δ*. These results suggest that Irc15 functions with Mrc1 in the cohesion pathway involving Ctf18–RFC. Finally, we compared the resumption of cell cycle progression of *irc15Δ* and the prefoldin mutants following a G2/M arrest. Although WT cells progressed through mitosis and started to enter G1 by 60 min, the majority of the *irc15Δ* and prefoldin mutant cells were still in mitosis at that time, showing a clear delay in cell cycle progression (Fig. 4F), consistent with a sister chromatid cohesion defect (Sonoda et al., 2001). Thus, we reveal that Irc15 and the prefoldin complex promote efficient sister chromatid cohesion. While Irc15 promotes this process, likely by facilitating the loading of centromeric cohesin, it is unclear how the prefoldin complex would affect this process. Given that prefoldin delivers unfolded proteins to cytosolic chaperonins (Vainberg et al., 1998), we checked whether it may affect the stability of the cohesin core subunits. However, Smc1, Smc3, Scc1 and Scc3 stability remained unaffected in *gim3Δ* cells (Fig. S4).

## DISCUSSION

Here, we generated a comprehensive genetic interaction network centered on cohesin comprising 373 genetic interactions specific for cohesin factors. The network uncovered novel connections for cohesin genes in various cellular processes. Moreover, it also revealed new factors involved in sister chromatid cohesion, namely the microtubule-associated protein Irc15 and the prefoldin complex members Gim3, Gim4 and Yke2. Thus, our genetic interaction map provides a unique resource for the further identification and functional interrogation of cohesin proteins.

Irc15 was initially identified in different screens that were designed to identify factors involved in chromosome segregation and DNA repair (Alvaro et al., 2007; Measday et al., 2005; Daniel et al., 2006; Jordan et al., 2007). It was also shown that Irc15 associates with microtubules, regulating their dynamics and mediating tension between kinetochores (Keyes and Burke, 2009). Here, we identified a novel role for Irc15 in centromeric cohesin loading and cohesion establishment. Proper centromeric cohesion is a prerequisite to generate a dynamic tension between microtubules and sister chromatids in yeast (Goshima and Yanagida, 2000; He et al., 2000; Tanaka et al., 2000). This tension is also required for the establishment of stable microtubule–kinetochore attachments (Ault and Nicklas, 1989; Nicklas and Ward, 1994; Koshland et al., 1988; Skibbens et al., 1995). Indeed, loss of Scc1 impairs both sister chromatid cohesion and kinetochore function in higher eukaryotes (Sonoda et al., 2001). However, in the case of *irc15Δ* our results suggest that the kinetochore defect did not result from the cohesin loading defect observed in this mutant background. Conversely, several inner and central kinetochore proteins play a role in the recruitment of pericentromeric cohesin (Eckert et al., 2007; Hinshaw et al., 2017). However, cells with defective microtubule–kinetochore attachments exhibit high levels of Scc1 loading at centromeres (Eckert et al., 2007). Given that Irc15 controls tension between kinetochores and microtubules (Keyes and Burke, 2009), and that we observed a decrease in centromeric cohesin loading in the absence of *IRC15*, it is unlikely that the cohesion defect in *irc15Δ* cells stems from a kinetochore defect. Rather, Irc15 may play independent roles in cohesin loading and microtubule–kinetochore attachment at centromeres.

We also identified the prefoldin complex as a new factor involved in sister chromatid cohesion. The prefoldin complex is a multi-subunit chaperone that assists in the proper folding of proteins in the cytosol (Vainberg et al., 1998). Even though it did not affect the stability of the cohesin core subunits, it is tempting to speculate that prefoldin targets one or more (other) factors involved in sister chromatid cohesion, thereby affecting this process. Alternatively, the involvement of the prefoldin complex in cohesion might also be related to its role in regulating chromatin structure during transcription elongation (Millan-Zambrano et al., 2013). To this end, it may either influence the transcription of genes involved in cohesion or allow the loading of the cohesin complex by generating nucleosome-free regions at transcribed genes (Millan-Zambrano et al., 2013). This hypothesis is supported by our genetic interaction network, which identified a strong relationship between cohesin factors and factors involved in gene expression and/or chromatin remodeling. To this end, it is interesting to note that the RSC chromatin remodeling complex facilitates the association of cohesin on chromosome arms by generating a nucleosome-free region (Huang et al., 2004; Huang and Laurent, 2004; Munoz et al., 2019). Moreover, the SWR1 complex deposits the histone variant H2A.Z, whose acetylation helps to maintain sister chromatin cohesion (Sharma et al., 2013). Finally, it was also shown that the NAD^+^-dependent deacetylase Hst3, a member of the sirtuin superfamily, is involved in sister chromatid cohesion through the acetylation of histone H3 at lysine K56 (Thaminy et al., 2007), and that strains harboring mutations in cohesin genes are sensitive to sirtuin inhibitors (Choy et al., 2015). These findings may enforce a potential link between prefoldin and chromatin remodeling in cohesion establishment.

Among the novel connections for cohesin genes, we identified several interactions linked to post-replication repair and nucleotide excision repair. Further studies may reveal the functional importance of the link between sister chromatid cohesion and these processes. Since defects in nucleotide excision repair are associated with Cockayne syndrome and xeroderma pigmentosum, we anticipate that the link between cohesin factors and this repair process may be relevant for disease etiology. In line with this, it was recently shown that the nucleotide excision repair structure-specific endonuclease ERCC1–XPF complex interacts with the cohesin complex and other proteins at promoters to silence imprinted genes during development in mice (Chatzinikolaou et al., 2017). Moreover, since sister chromatid cohesion and the factors involved are well conserved from yeast to men (Xiong and Gerton, 2010), our network may also inform on genetic interactions of cohesin factors mutated in cohesinopathies or cancer.

## MATERIALS AND METHODS

### Genetic interaction map analysis

The genetic interaction map was generated and analyzed as previously described (Srivas et al., 2013). Briefly, an array of 1494 genes (Table S2) was collected from the yeast deletion collection (Mat-alpha) and the DAmP library containing a KANMX selection marker. To generate the query genes (Table S1), mutant strains carrying deletion mutations were generated by PCR gene targeting (Longtine et al., 1998), while mutants carrying point mutations were either generated using the MIRAGE method (Nair and Zhao, 2009) in a strain containing synthetic genetic array (SGA) anti-diploid selection markers and a NATMX selection marker, or by using strains obtained from Charles Boone (Donnelly Centre, University of Toronto, Canada) and Philip Hieter (Michael Smith Laboratories, University of British Columbia, Canada). Primers used to generate these mutants are available upon request. Owing to the presence of temperature-sensitive mutants, the generation of double mutants was performed at permissive temperature (23°C) with use of the SGA procedure in quadruplicate using the ROTOR HDA (Singer Instruments) pinning robot (Tong and Boone, 2006). Genetic interactions were assessed at semi-permissive temperature (30°C). Pictures were taken with a Canon Powershot G3. Colony sizes were quantified and normalized using Matlab Colony Analyzer (Bean et al., 2014). Quantitative S-scores were calculated using Matlab as previously described (Collins et al., 2010; Guenole et al., 2013). Network visualizations of genetic interactions were performed using Cytoscape (Shannon et al., 2003). The Cytoscape plugin BiNGO was used for GO term enrichment analysis (Maere et al., 2005). Unsupervised clustering was performed using Cluster 3.0 using a selection of array genes that show a magnitude of S-score>2.0 in at least one of the query genes and a variation with a standard deviation >0.8 in the query genes. The clustering was visualized in a heatmap using Java TreeView.

### Yeast strains and culture conditions

A strain expressing 18Myc-tagged Scc1 and HA-tagged Pds1 was used in flow cytometry and Scc1-based ChIP experiments. PCR gene targeting was used to generate the tagged alleles and gene deletions (Table S8). A strain carrying a LacO array integrated on chromosome IV 10 kb away from *CEN4* and expressing a LacR–GFP fusion protein was used for sister chromatid cohesion assays (Shimada and Gasser, 2007). PCR gene targeting was used to generate gene deletions in this background (Table S8). Primers used to generate yeast strains are available upon request. All yeast strains were cultured in rich YPAD medium or Synthetic Complete medium lacking methionine (SC-methionine).

### Chromatin immunoprecipitation

Chromatin immunoprecipitation (ChIP) was performed as previously described with slight modifications (Cobb et al., 2003). Briefly, cells were grown to 5×10^6^ cells/ml in YPAD and synchronized in G2/M by incubation with nocodazole (7.5 µg/ml) for 2 h for Scc1 ChIP. Nocodazole (7.5 µg/ml) was added a second time after 1 h of incubation. Alternatively, cells were synchronized in G1 with α-factor for 2 h, washed and released in YPAD containing nocodazole for 0, 30, 60, 90 and 120 min. Samples were fixed with 1% formaldehyde. For Ndc80–GFP ChIP, cells were grown overnight in SC-methionine containing 2% raffinose, diluted and grown in the presence of 2% glucose or 2% galactose for 4 h, diluted to 5×10^6^ cells/ml and fixed with 1% formaldehyde. Extracts were prepared in lysis buffer (50 mM Hepes, pH 7.5, 140 mM NaCl, 1 mM Na EDTA, 1% Triton X-100 and 0.1% sodium deoxycholate) containing protease inhibitors. Extracts were subjected to immunoprecipitation with Dynabeads mouse or rabbit IgG (Invitrogen, M-280) coated with antibody against c-Myc (9B11, Cell Signaling) or GFP (ab290, Abcam). DNA was purified and enrichment at specific loci was measured by performing quantitative (q)PCR. Relative enrichment was determined by the 2^−ΔΔCt^ method (Livak and Schmittgen, 2001; Cobb and van Attikum, 2010). Dynabeads alone were used to correct for background. An amplicon 11 kb downstream of ARS305, devoid of Scc1 binding, was used for Scc1 ChIP normalization (Tittel-Elmer et al., 2012). An amplicon devoid of Ndc80 binding (Neg1p1) was used for Ndc80 ChIP normalization (Lefrancois et al., 2013). Primers used are listed in Table S9.

### Sister chromatid cohesion assay

Sister chromatid cohesion was assayed using a strain containing a LacO repeat integrated at chromosome 4 between *ARS1* and *CEN4* at 10 kb distance to *CEN4* and a LacR–GFP expression cassette integrated at the *HIS3* locus (Shimada and Gasser, 2007). Cells were grown to mid-log phase in YPAD, synchronized in G1 by incubation with α-factor for 1.5 h, or in G2/M by incubation with nocodazole (15 µg/ml) for 1 h. Cells were fixed in 4% paraformaldehyde at room temperature for 15 min, washed and resuspended in KPO_4_/Sorbitol solution (10 mM KPO_4_, 1.2 M Sorbitol, pH 7.5). Images of cells were acquired on a Zeiss AxioImager M2 widefield fluorescence microscope equipped with 100× PLAN APO (1.4 NA) oil-immersion objectives (Zeiss) and an HXP 120 metal-halide lamp used for excitation. Fluorescence signals were detected using the following filters: GFP/YFP 488 (excitation filter: 470/40 nm, dichroic mirror: 495 nm, emission filter: 525/50 nm). Images were recorded and analyzed using ZEN 2012 software.

### Flow cytometry

Cells were grown to midlog phase in YPAD, synchronized in G1 by incubation with α-factor for 1.5 h, or in G2/M by incubation with nocodazole (15 µg/ml) for 1 h. Alternatively, cells were grown to midlog phase in YPAD, synchronized in G2/M by incubation with nocodazole (15 µg/ml) for 2 h, washed and released in YPAD. Samples were prepared as previously described (Haase and Lew, 1997). Data were acquired on a BD FACSCalibur (BD Biosciences) or on a Novocyte (ACEA Biosciences, Inc) and analyzed with FlowJo or NovoExpress software, respectively.

### Spot dilution test

Cells were grown overnight in YPAD and then plated in fivefold serial dilutions starting at a density of 6×10^6^ cells/ml (OD_600_ nm=0.5) on YPAD plates. Cells were grown for 3 days at the semi-permissive temperature (30°C) before images were taken.

### Cycloheximide chase experiment

Cells expressing Scc1–18Myc, Scc3–6FLAG, Smc1–6FLAG or Smc3–6FLAG (Table S8) were subjected to cycloheximide chase analysis as previously described (Buchanan et al., 2016). Samples were collected at 0, 30, 60 and 90 min after cycloheximide treatment. Whole-cell extracts were prepared by post-alkaline protein extraction and analyzed by SDS-PAGE. Western blotting was performed using an anti-c-Myc antibody (1:1000, 9E10, cat. no. sc-40, Santa Cruz Biotechnology) and FLAG antibody (1:5000, clone M2, cat. no. F1804, Sigma). Ponceau staining served as a loading control.

### Curation of *S. cerevisiae*–*S. pombe* and *S. cerevisiae–H. sapiens* orthologs

Information about budding yeast-to-human and budding yeast-to-fission yeast orthologs was collected from two different sources, InParanoid (O'Brien et al., 2005) and PomBase (Lock et al., 2018), and is presented in Table S7. InParanoid inventories orthologs based on protein sequence similarity, whereas PomBase curates orthologs based on both function and sequence similarity.

## Supplementary Material

Supplementary information

Reviewer comments
