# Supplementary material for: A genetic interaction map centered on cohesin reveals auxiliary factors involved in sister chromatid cohesion in S. cerevisiae
Source: J Cell Sci. 2020 May 22;133(10):jcs237628. doi: 10.1242/jcs.237628 (PMC7325435; doi:10.1242/jcs.237628)
Supplement: Supplementary information [file joces-133-237628-s1.pdf]

Sun et al_Figure S1

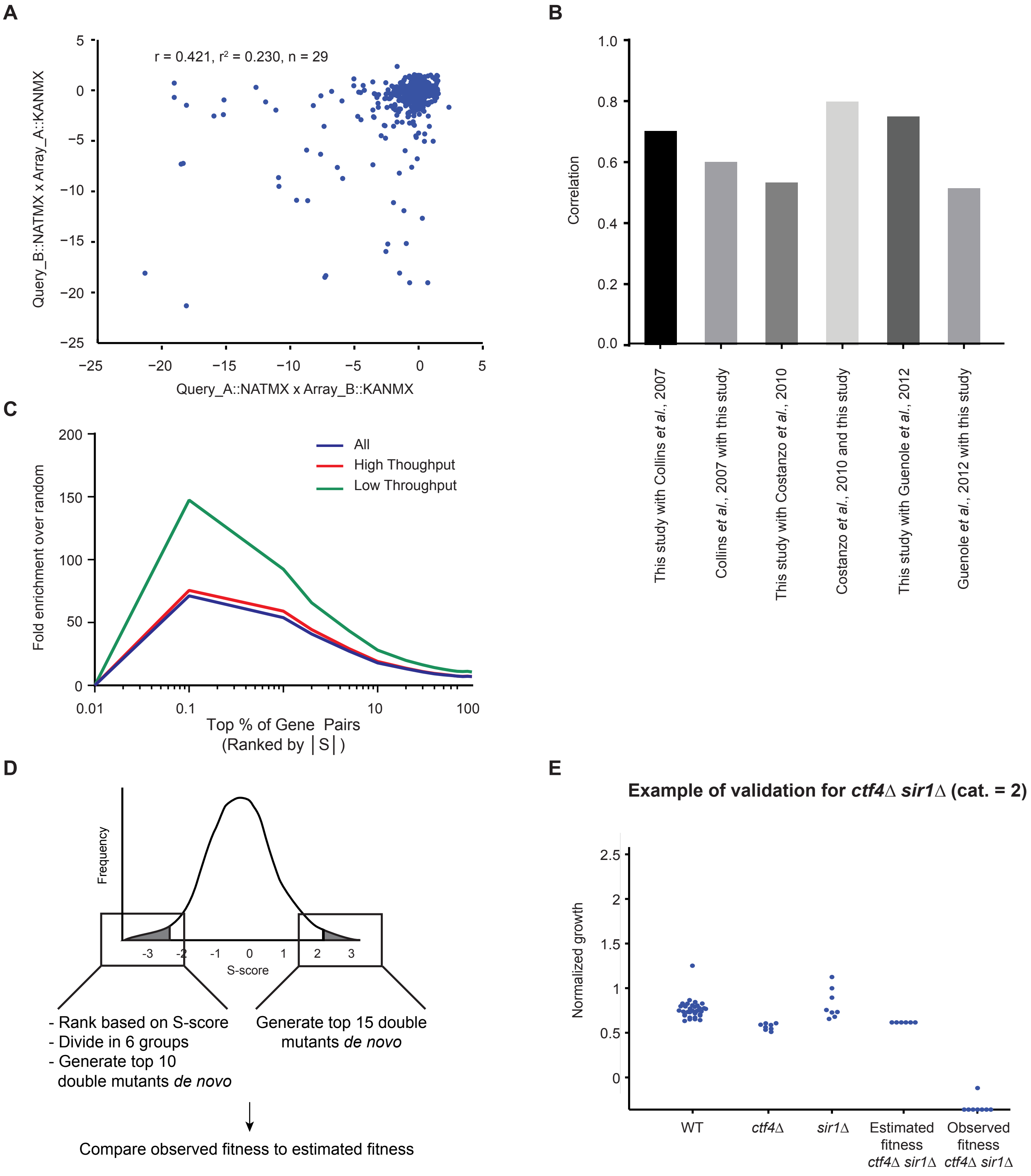

F

Category	Double mutants tested	Genetic interaction validated (Padjust<0.05)
1	7	4
2	10	10
3	10	8
4	10	5
5	9	7
6	10	8
Pos	15	7
Total	71	49

G

	P_{adjust} <0.05
FDR negative interactions	25.0%
FDR positive interactions	53.3%
FDR average	31.0%

Figure S1. Quality control of the genetic interaction map

(A) Correlation of S-scores from genetic interactions in double mutants generated by reciprocal crossings of query and array strains: query *gene A::NATMX x* array *gene B::KANMX* and query *gene A::KANMX,* array *gene B::NATMX*.

(B) Correlation of genetic interaction scores of common interactions between this study and other studies.

(C) Fold enrichment for genetic interactions that are present in the Biogrid database (version 3.2; (Stark et al., 2006)) is shown. Fold enrichment is defined as n/r, where n is the number of highest scoring genetic interactions (x-axis) found in the Biogrid database, while r is the number of overlapping interactions expected at random.

(D) Outline of the validation of genetic interactions with S-scores ≥ 2 or S-scores ≤ -2.5). Negative interactions were ranked and divided in 6 groups. Double mutants corresponding to the top 10 interactions within each group were generated *de novo* in multitude using SGA technology (n>8). The fitness of double mutants was compared to the estimated fitness of the double mutants using t-test statistics and correction for multiple testing (Benjamini-Hochberg). Estimated fitness of the double mutant was calculated by combining fitness of the corresponding single mutants (Mani et al., 2008), which were also generated *de novo* by crossing them with WT dummy strains containing either a *his3::*KANMX or *his3::*NATMX allele. Positive interactions were validated in the same way, except that only the top 15 interactions were tested.

(E) Example of the outcome of the validation of the negative interaction between *CTF4* and *SIR1*.

(F) Table showing the outcome of the validation of 71 genetic interactions.

(G) Table showing the false discovery rates based on the validation of 71 genetic interactions.

Journal of Cell Science • Supplementary information

Sun et al_Figure S2

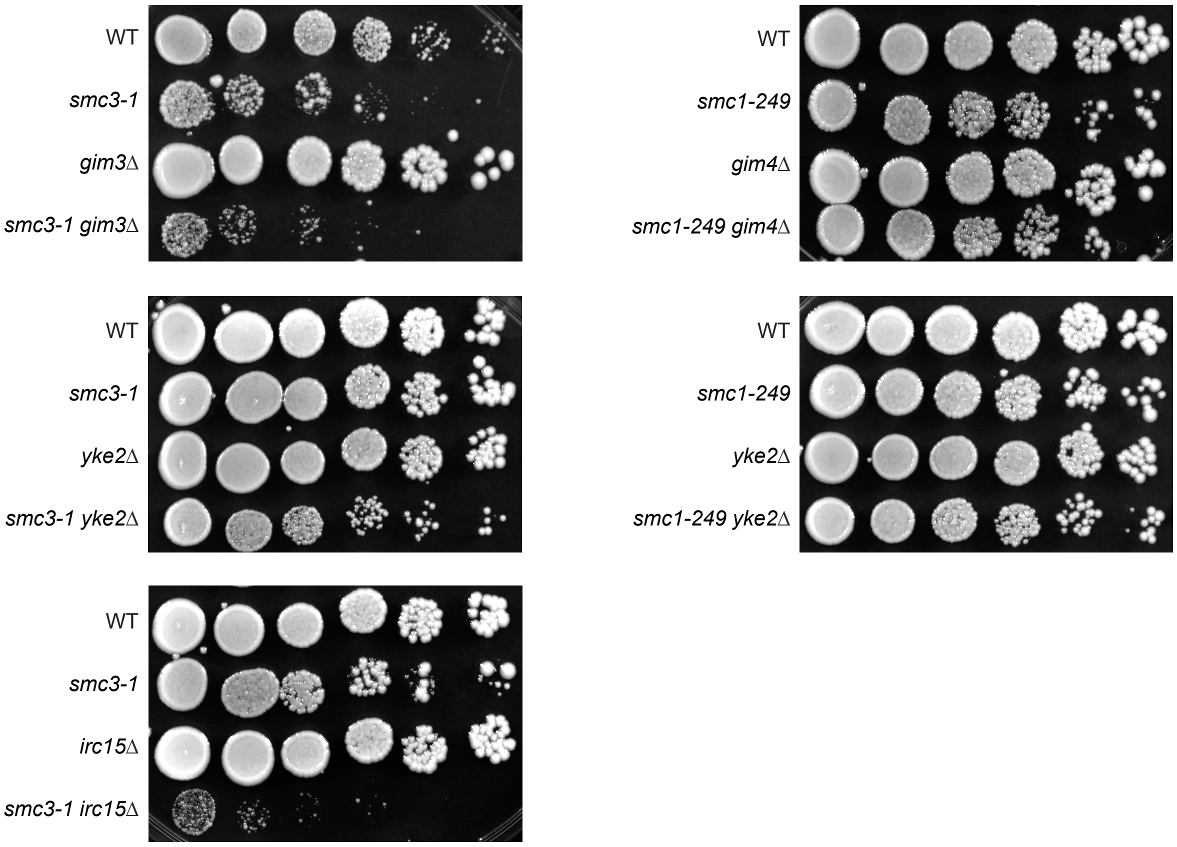

Figure S2. Validation of negative genetic interactions for *IRC15*, *GIM3*, *GIM4* and *YKE2*.

Drop assay for the indicated strains. Ten-fold serial dilutions of exponentially growing cells were spotted on rich medium and incubated at semi-permissive temperature (30°C).

Sun et al_Figure S3

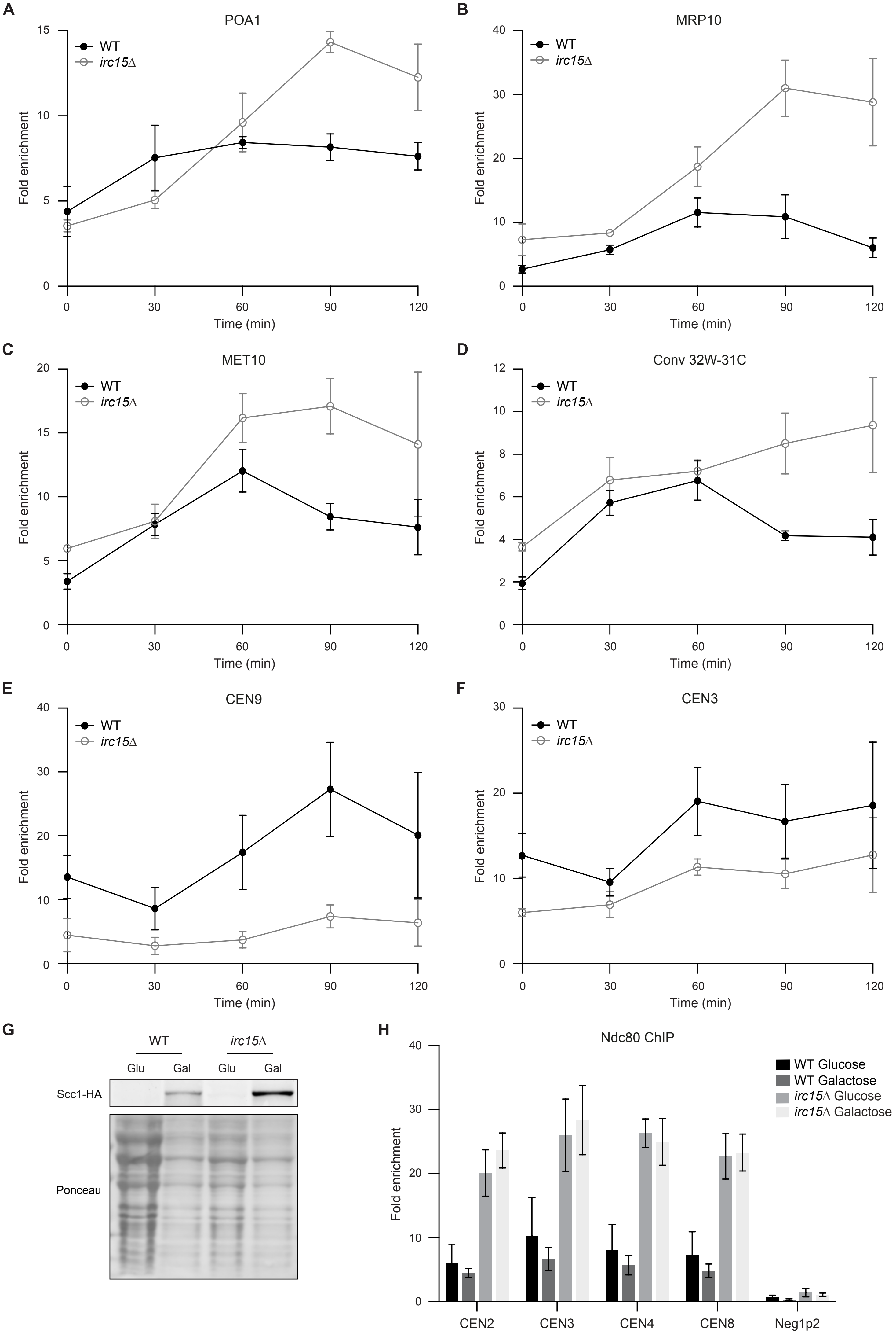

Journal of Cell Science • Supplementary information

Figure S3. Cohesion defects in *irc15Δ* do not stem from cohesin translocation from centromeres to chromosome arms and do not result in kinetochore defects.

(A-F) Enrichment of Scc1-Myc assessed by ChIP-qPCR at *POA1* (A), *MRP10* (B), *MET10* (C), *Conv 32W-31C* (D), *CEN9* (E) and *CEN3* (F) in WT and *irc15Δ* cells. Strains were arrested in G1 and released in nocodazole. Enrichment corresponds to the ratio of the Scc1-Myc signal over beads alone. Average enrichment with standard error of the mean of 3 independent experiments is shown.

(G) Western blot analysis of Scc1-HA expression after incubation in medium with galactose (Gal; Scc1-HA overexpression) or glucose (Glu; Scc1-HA repression).

(H) Enrichment of Ndc80-GFP assessed by ChIP-qPCR at four centromeres in the indicated strains in presence of glucose (Scc1-HA repression) or galactose (Scc1-HA overexpression). Enrichment corresponds to the ratio of the Ndc80-GFP signal at CEN2, CEN3, CEN4, CEN8 and Neg1p2 over Neg1p1 in GFP IPs over IPs with beads alone. Average enrichment with standard error of the mean of 2 (glucose) or 3 (galactose) independent experiments is shown.

Journal of Cell Science • Supplementary information

Sun et al_Figure S4

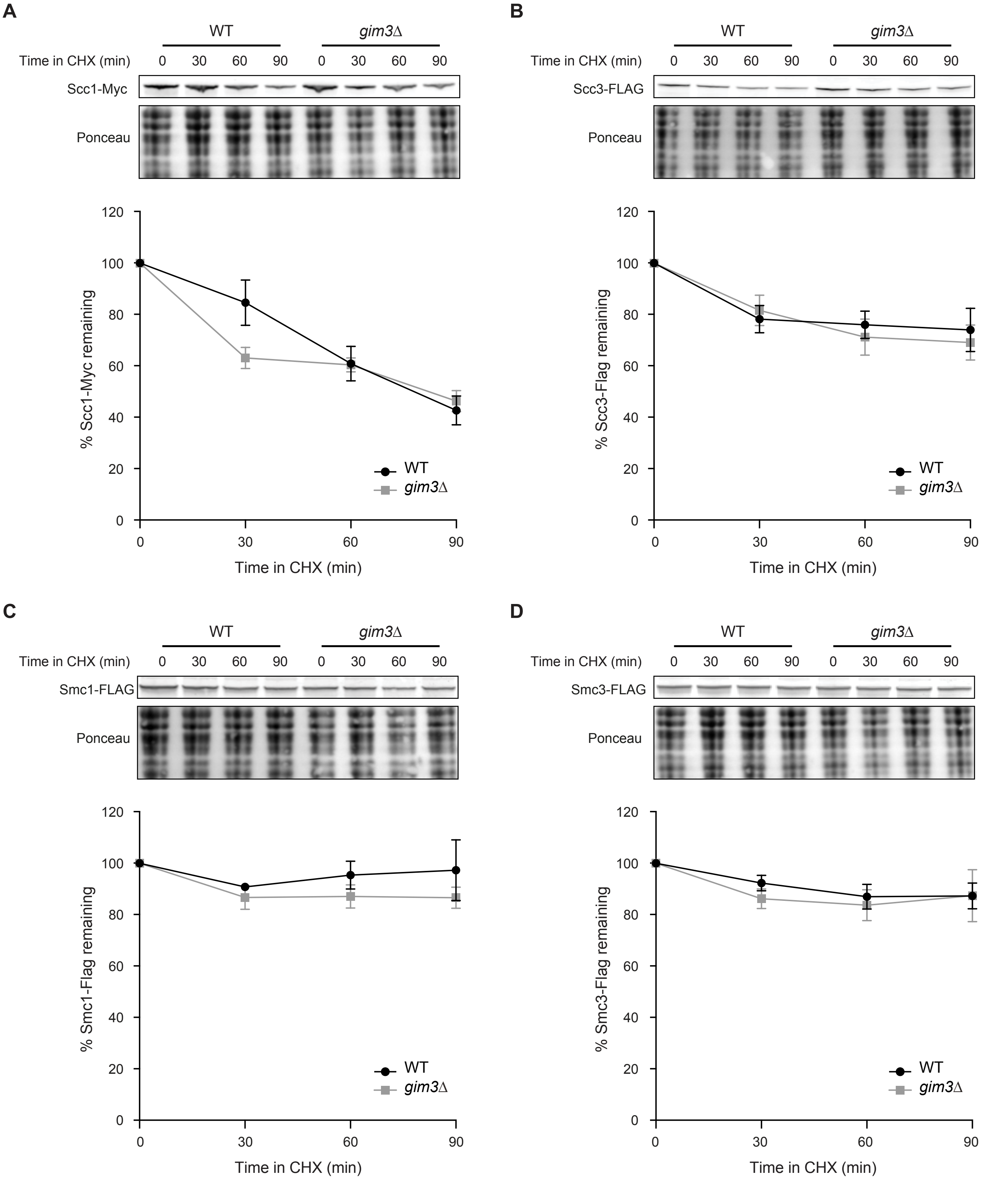

Figure S4. Cohesin core subunits stability is not affected in *gim3Δ* cells.

(A-D) Western blot analysis and quantification of Scc1-Myc (A), Scc3-Falg (B), Smc1-Flag (C) and Smc3-Flag (D) levels in WT and *gim3Δ* cells. The indicated strains were subjected to cycloheximide chase analysis. Ponceau staining served as loading control. Signal intensities were adjusted to Ponceau staining. The ratio was set to 100% for the first time point for each strain and subsequent time points were normalized to it.

Journal of Cell Science • Supplementary information

Table S1: List of query genes

Click here to Download Table S1

Table S2: List of array genes

Click here to Download Table S2

Table S3: S-scores of the cohesin/DDR interaction map

Click here to Download Table S3

Table S4: Gene Ontology analysis of the cohesin/DDR interaction map

Click here to Download Table S4

Table S5: Gene Ontology analysis of cohesin-related genes

Click here to Download Table S5

Table S6: Gene Ontology analysis of DDR-related genes

Click here to Download Table S6

Table S7: Ortholog information for the cohesin interaction network

Click here to Download Table S7

Table S8: Yeast strains used in this study

Click here to Download Table S8

Table S9: List of primers used for qPCR

Click here to Download Table S9
