# Supplementary material for: A genetic interaction map centered on cohesin reveals auxiliary factors involved in sister chromatid cohesion in S. cerevisiae
Source: J Cell Sci. 2020 May 22;133(10):jcs237628. doi: 10.1242/jcs.237628 (PMC7325435; doi:10.1242/jcs.237628)
Supplement: Reviewer comments [file joces-133-237628_review_history.pdf]

Journal of Cell Science

A genetic interaction map centered on cohesin reveals auxiliary factors involved in sister chromatid cohesion in *S. cerevisiae*

Su Ming Sun, Amandine Batte, Mireille Elmer, Sophie C. van der Horst, Tibor van Welsem, Gordon Bean, Trey Ideker, Fred van Leeuwen and Haico van Attikum
DOI: 10.1242/jcs.237628

Editor: David Glover

Review timeline
Original submission: 7 August 2019
Editorial decision: 16 September 2019
First revision received: 17 February 2020
Accepted: 26 March 2020

Original submission

<u>First decision letter</u>

MS ID#: JOCES/2019/237628

MS TITLE: A genetic interaction map centered on cohesin reveals auxiliary factors in sister chromatid cohesion

AUTHORS: Su Ming Sun, Amandine Batte, Mireille Tittel-Elmer, Tibor van Welsem, Gordon Bean, Trey Ideker, Fred van Leeuwen, and Haico van Attikum
ARTICLE TYPE: Short Report

We have now reached a decision on the above manuscript.

To see the reviewers' reports and a copy of this decision letter, please go to: https://submit-jcs.biologists.org and click on the 'Manuscripts with Decisions' queue in the Author Area. (Corresponding author only has access to reviews.)

As you will see, the reviewers raise a number of substantial criticisms that prevent me from accepting the paper at this stage. As you see, one is more in favour and one more against. On balance, I feel that the broader insight given by your study is valuable and suitable for the journal. If you think that you can deal satisfactorily with the criticisms on revision, I would be pleased to see a revised manuscript. We would then return it to the reviewers and also try and find one additional referee.

Please ensure that you clearly highlight all changes made in the revised manuscript. Please avoid using 'Tracked changes' in Word files as these are lost in PDF conversion.

I should be grateful if you would also provide a point-by-point response detailing how you have dealt with the points raised by the reviewers in the 'Response to Reviewers' box. Please attend to all of the reviewers' comments. If you do not agree with any of their criticisms or suggestions please explain clearly why this is so.

© 2020. Published by The Company of Biologists under the terms of the Creative Commons Attribution License (https://creativecommons.org/licenses/by/4.0/).

Reviewer 1

Advance summary and potential significance to field

In the manuscript, Sue et al. map genetic interactions of 17 cohesin and 18 DDR genes. Genetic interactions were determined by normalized colony size.
The authors identified hundreds of negative and tens of positive interactions for these gene sets. The screen revealed previously reported interactions, as well as newly identified interactions. The authors selected two of these new genes for further investigation- the prefoldin complex and IRC15. They used ChIP and 2 dots cohesion assay to show that prefoldin affect cohesion establishment and Irc15 affects centromeric cohesion. While some of the genetic data is novel, the mechanism is not fully addressed and the general contribution to the field is mild. Some experiments need to be redesigned in order to fully support the conclusions.

Comments for the author

1. Statistics is needed in fig 3C. Is the increase of Scc1 on chromosome arm is significant? The authors should at least discuss this observation. One possible explanation is that the defective centromere assembly in irc15delta cell induces cohesin translocation from the centromere to the arms. This can be tested by a ChIP time course from G1 to G2/M.
2. It is not clear if the reduced Scc1 levels in irc15delta cells is affected by the problem with kinetochore assembly or vice versa.
3. If irc15delta induce a segregation defect one would expect cells with >2n DNA content rather than mitotic delay. Did the authors observe aneuploidy? The cell cytometry results suggest on a microtubules attachment defect rather than a cohesion defect.
4. The interaction with prefoldin required careful examination of protein stability. The experiment in fig S3 is not sufficient. The stability of cohesin core subunits: SMCs, Scc1 and Scc3 needs to be assessed in a cycloheximide chase experiment. Ideally, nuclear/cytoplasmic localization should be determined.
5. The effect of double and triple mutation in prefoldin genes on cohesion should be tested in order to determine if the holocomplex is involved. It would also be interesting to test if the irc1delta is epistatic with the two known non-essential cohesion pathways or define a new pathway (Xu, Boone and Brown, Genetics, 2007).
6. The work would have benefit from a more profound discussion.

Reviewer 2

Advance summary and potential significance to field

Su Ming Sun et al. systematically mapped genetic interactions of 17 cohesin-related genes centered on quantitative growth measurements of >52,000 gene pairs in budding yeast. It unveiled a cohesin functional map and discovered novel involvements of microtubule-associated protein Irc15 and the prefoldin complex members in sister chromatid cohesion. The screen is comprehensive, therefore is helpful to understand cohesin interactome. The new auxiliary factors of cohesin discovered in this study provide new mechanisms regulating sister-chromatid cohesion.

Comments for the author

In general the manuscript is well written and the results are clearly presently. The involvement of microtubule-associated protein Irc15 and the prefoldin complex members in sister chromatid cohesion is new and interesting, although further works are need understand the mechanism in cohesin loading.

Comments:
1. In Figure 1C, the GO enrichment (P-value) of 'cohesin/DDR-related interactions' and 'Cohesin-related interactions' are same. Please confirm if there is a mistake in using chart. In addition, 'Sisterchromatid cohesion' should be changed to 'Sister chromatid cohesion'.

© 2020. Published by The Company of Biologists under the terms of the Creative Commons Attribution License (https://creativecommons.org/licenses/by/4.0/).

2. It will be very helpful for researchers using other organisms (such as fission yeast) to understand the interactions, if author can provide homolog informations for the interaction described in Figure 2 as supplementary information (such as a excel file).
3. Since overall false discovery rate is high (31%), verification of the negative interactions highlighted in Figure 3A (such as interaction of smc1-249/smc3-1 with irc15, gim3, gim4, yke2 deletion mutants) might be required. In addition, showing spot test results will be helpful for audience to judge how strong the interactions are.
4. In Figure 4C, it's clear in G2/M arrested condition that more cells in mutants (gim3, gim4, yke2 and pac10 deletion mutants) than wild type have more than one GFP spot, which suggests a defect in sister chromatid cohesion. However, it's also clear that in G1 arrested condition, more cells in these mutants than wild type have more than one spot. Is there any reasonable explanation?
5. There's no numbering in the manuscript (pdf), therefore it's not easy to point out. It is reported that cohesin has a role in DNA damage repair (Nagao K et al. Nature. 2004; McAleenan A et al. Nature. 2013). In page 6, Author described 'For example, several interactions between cohesin factors and genes involved in nucleotide excision repair, such as RAD16 and RAD1 with SMC1 and RAD10 with RAD61, in mismatch repair such as MSH2 with MDC1 and RAD61, or in template switching, such as RAD5 with DCC1 and RMI1, might indicate a novel role for cohesin in post-replication repair' without any citation of previous reports.
6. In page 8. The evidences to support the subtitle 'The prefoldin complex regulates cohesion establishment' is too weak, therefore revision of this subtitle is required.

First revision

<u>Author response to reviewers' comments</u>

<u>Point-by-point response to the reviewer's comments</u>

MS ID#: JOCES/2019/237628

A genetic interaction map centered on cohesin reveals auxiliary factors in sister chromatid cohesion

Su Ming Sun, Amandine Batte, Mireille Tittel-Elmer, Sophie van der Horst, Tibor van Welsem, Gordon Bean, Trey Ideker, Fred van Leeuwen, and Haico van Attikum

We would like to thank the reviewers for their positive feedback and constructive comments on our manuscript. Based on this we have performed several additional experiments, which were added to the manuscript. The results of these experiments fully support and extend the conclusions of our work. Below you will find a point-by-point response to the comments (text in red). All changes to the manuscript are also indicated with text in red.

Reviewer 1

Reviewer 1 Advance Summary and Potential Significance to Field:

In the manuscript, Sue et al. map genetic interactions of 17 cohesin and 18 DDR genes. Genetic interactions were determined by normalized colony size. The authors identified hundreds of negative and tens of positive interactions for these gene sets. The screen revealed previously reported interactions, as well as newly identified interactions. The authors selected two of these new genes for further investigation- the prefoldin complex and IRC15. They used ChIP and 2 dots cohesion assay to show that prefoldin affect cohesion establishment and Irc15 affects centromeric cohesion. While some of the genetic data is novel, the mechanism is not fully addressed and the general contribution to the field is mild. Some experiments need to be redesigned in order to fully support the conclusions.

© 2020. Published by The Company of Biologists under the terms of the Creative Commons Attribution License (https://creativecommons.org/licenses/by/4.0/).

Reviewer 1 Comments for the Author:

1. Statistics is needed in fig 3C. Is the increase of Scc1 on chromosome arm is significant? The authors should at least discuss this observation. One possible explanation is that the defective centromere assembly in irc15delta cell induces cohesin translocation from the centromere to the arms. This can be tested by a ChIP time course from G1 to G2/M.

The ChIP originally presented in Fig. 3C was repeated two more times. Average enrichment with standard error of the mean of four independent experiments is now shown in the new figure (see Fig. 3C). Statistics were added using an unpaired t-test. The new data show that the increase of Scc1 on chromosome arms in the absence of *IRC15* is significant. This observation is discussed on page 7 of the revised manuscript.

As suggested by the reviewer, we also assessed Scc1 recruitment by ChIP during a time course from G1 to G2/M in WT and *irc15Δ* cells. Cells were arrested in G1-phase by alpha- factor, washed and released in nocodazole. Samples were taken at 0, 30, 60, 90 and 120 minutes after release. Particularly at 90 and 120 minutes after release, we observed an increase in Scc1 recruitment at chromosome arms and a decrease at centromeres in *irc15Δ* when compared to WT (see new Fig. S4), corroborating our previous results (see Fig. 3C). Importantly, however, we did not observe a translocation of Scc1 from the centromeres to the chromosome arms in *irc15Δ* cells during the course of the experiment. The new data are presented in Fig. S3A-F and discussed on page 7 of the revised manuscript.

2. It is not clear if the reduced Scc1 levels in irc15delta cells is affected by the problem with kinetochore assembly or vice versa.

To address this point, we have examined whether overexpression of Scc1 could rescue the problem with kinetochore assembly in *irc15* strains. To this end, we have GFP-tagged Ndc80 in the kinetochore-associated Ndc80 complex, which is involved in kinetochore assembly, in WT and *irc15Δ* strains carrying a galactose-inducible allele of Scc1. Subsequently, ChIP experiments were performed to monitor kinetochore assembly in these strains either with (in medium containing galactose) or without (in medium containing glucose) Scc1 overexpression (see Fig. S3G). We observed comparable levels of Ndc80-GFP occupancy at 4 different centromeres (CEN2, CEN3, CEN4 and CEN8) in WT cells in which Scc1 was overexpressed or not, while the negative control locus was devoid of Ndc80 as reported previously (Lefrançois et al., *Plos Genet.*, 2013) (see Fig. S3H). However, a 4-fold increase in Ndc80-GFP binding at these centromeres was observed in *irc15Δ* cells when Scc1 was not overexpressed, which is indicative of a kinetochore assembly defect. Importantly, similar levels of Ndc80-GFP occupancy were observed when Scc1 was overexpressed in these cells (see Fig. S3H), suggesting that reduced Scc1 levels at centromeres do not cause kinetochore problems in *irc15Δ* cells. This is in line with a previous report showing that Irc15 is a microtubule- associated protein that maintains proper kinetochore organization by regulating microtubule dynamics (Keyes and Burke, Curr. Biol., 2009). The new data are presented in Fig. S3G-H and discussed on page 7-8 of the revised manuscript.
Irc15 regulates the tension between microtubules and kinetochores (Keyes and Burke, *Curr. Biol.*, 2009) and in cells lacking tension at kinetochores higher levels of Scc1 binding at pericentromeric regions were observed (Eckert *et al.*, Genes Dev., 2007). However, we show a decrease in the binding of Scc1 at centromeres in *irc15Δ* cells (see Fig. 3C). This suggests that the cohesion defect in *irc15Δ* cells does not stem from a kinetochore defect. This point is discussed on page 10 of the revised manuscript.

3. If irc15delta induce a segregation defect one would expect cells with >2n DNA content rather than mitotic delay. Did the authors observe aneuploidy? The cell cytometry results suggest on a microtubules attachment defect rather than a cohesion defect.

Flowcytometry or FACS analysis of WT and *irc15Δ* cells from an asynchronous population was already shown in Fig. 4C (bottom) and no aneuploidy was detected. When we repeated this FACS analysis, similar results were obtained (see Reviewer only Figure 1). Thus, the normal ploidy of *irc15Δ* cells indicates that there is no segregation defect following Irc15 loss, and that the mitotic delay (Fig. 4F) indeed results from a cohesion defect rather than a microtubule attachment defect.

© 2020. Published by The Company of Biologists under the terms of the Creative Commons Attribution License (https://creativecommons.org/licenses/by/4.0/).

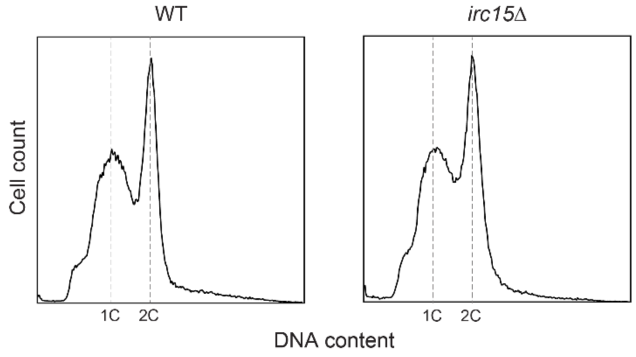

Reviewer only Figure 1.

Flow cytometry analysis of asynchronous populations of WT and *irc15*Δ cells.

4. The interaction with prefoldin required careful examination of protein stability. The experiment in fig S3 is not sufficient. The stability of cohesin core subunits: SMCs, Scc1 and Scc3 needs to be assessed in a cycloheximide chase experiment. Ideally, nuclear/cytoplasmic localization should be determined.

The stability of Scc1-Myc, Scc3-FLAG, Smc1-FLAG and Smc3-Flag were assessed in WT and *gim3Δ* cells following a cycloheximide chase for 0, 30, 60 or 90 minutes. Scc1 was clearly degraded during the course of the experiment, whereas Scc3, Smc1 and Smc3 remained rather stable. Importantly, protein stability was comparable in WT and *gim3Δ* cells, indicating that the stability of the Scc1, Scc3, Smc1 and Sm3 is not regulated by prefoldin. Consequently, we did not examine nuclear/cytoplasmic localization of these cohesin core subunits in WT and *gim3Δ* cells. The new results are presented in Fig. S4 and discussed on page 9 of the revised manuscript.

5. The effect of double and triple mutation in prefoldin genes on cohesion should be tested in order to determine if the holocomplex is involved. It would also be interesting to test if the irc15delta is epistatic with the two known non-essential cohesion pathways or define a new pathway (Xu, Boone and Brown, Genetics, 2007).

We managed to generate a double mutant for two prefoldin genes, *GIM4* and *YKE2*. Although *gim4Δ yke2Δ* cells showed a severe growth defect and formed small colonies, we were able to assess sister chromatid cohesion in this strain using the LacO/LacR-GFP-based cohesion assay. We observed a cohesion defect in the *gim4Δ yke2Δ* double mutant, which was comparable to that in the *gim4Δ* and *yke2Δ* single mutants, suggesting that the holocomplex is involved in sister chromatid cohesion. This new result is presented in Fig. 4D and discussed on page 9 of the revised manuscript. Of note, we were unsuccessful in generating a *gim3Δ yke2Δ* double mutant, suggesting that the combined loss of *GIM3* and *YKE2* is lethal. Therefore, and because the *gim4Δ yke2Δ* mutants showed a severe growth defect, we did not generate a triple mutant for prefoldin genes.

Epistasis analysis was performed to assess whether *IRC15* is part of the *CHL1*- or *MRC1*- dependent cohesion pathway (Xu *et al.*, Genetics, 2007). To this end, we generated in *irc15Δ chl1Δ and irc15Δ mrc1Δ* strains and examined sister chromatid cohesion in these strains using the LacO/LacR-GFP-based cohesion assay. Interestingly, *irc15Δ* was epistatic with *mrc1Δ*, but additive with *chl1Δ*, indicating that *IRC15* is part of the *MRC1*-dependent cohesion pathway. These new results are presented in Fig. 4E and discussed on page 8-9 of the revised manuscript.

6. The work would have benefit from a more profound discussion.

The manuscript was initially submitted as a Short Report. With a word limit of 3000 words and a combined Results & Discussion section, we were unable to provide a profound discussion. However, because a substantial amount of additional work has been added to our revised manuscript, the editor agreed to submit the manuscript as a Research Article. This allowed us to include a separate and more extensive discussion. The new Discussion section can be found on page 9-11of the revised manuscript.

© 2020. Published by The Company of Biologists under the terms of the Creative Commons Attribution License (https://creativecommons.org/licenses/by/4.0/).

Reviewer 2

Reviewer 2 Advance Summary and Potential Significance to Field:

Su Ming Sun et al. systematically mapped genetic interactions of 17 cohesin-related genes centered on quantitative growth measurements of >52,000 gene pairs in budding yeast. It unveiled a cohesin functional map and discovered novel involvements of microtubule- associated protein Irc15 and the prefoldin complex members in sister chromatid cohesion. The screen is comprehensive, therefore is helpful to understand cohesin interactome. The new auxiliary factors of cohesin discovered in this study provide new mechanisms regulating sister-chromatid cohesion.

Reviewer 2 Comments for the Author:

In general the manuscript is well written and the results are clearly presently. The involvement of microtubule-associated protein Irc15 and the prefoldin complex members in sister chromatid cohesion is new and interesting, although further works are need understand the mechanism in cohesin loading.

Comments:
1. In Figure 1C, the GO enrichment (P-value) of 'cohesin/DDR-related interactions' and 'Cohesin-related interactions' are same. Please confirm if there is a mistake in using chart. In addition, 'Sisterchromatid cohesion' should be changed to 'Sister chromatid cohesion'.

There was indeed a mistake in the chart for the 'Cohesin-related interactions'. The correct GO enrichment (P-value) is now presented in the new version of Fig. 1C. In this figure, we also changed 'Sisterchromatid cohesion' to 'Sister chromatid cohesion'.

2. It will be very helpful for researchers using other organisms (such as fission yeast) to understand the interactions, if author can provide homolog informations for the interaction described in Figure 2 as supplementary information (such as a excel file).

We now provide information for orthologous genes in fission yeast and human for the interactions presented in Fig. 2 as supplementary information (see new Table S7) and mention this information on page 5 of the revised manuscript. Information about the curation of the orthologs genes is detailed in the Material & Methods section.

3. Since overall false discovery rate is high (31%), verification of the negative interactions highlighted in Figure 3A (such as interaction of smc1-249/smc3-1 with irc15, gim3, gim4, yke2 deletion mutants) might be required. In addition, showing spot test results will be helpful for audience to judge how strong the interactions are.

smc1-249 and *smc3-1* mutants were crossed *de novo* with *irc15Δ, gim3Δ, gim4Δ* or *yke2Δ* mutants. From these crosses we were able to obtain *smc3-1 gim3Δ*, *smc3-1 yke2Δ*, *smc3-1 irc15Δ*, *smc1-249 gim4Δ* and *smc1-249 yke2Δ* double mutants. Spot dilution tests confirmed the negative genetic interactions for *smc3-1 gim3Δ*, *smc3-1 yke2Δ* , *smc3-1 irc15Δ*, *smc1-249 gim4Δ* and *smc1-249 yke2Δ*. Interactions between *smc3-1* and *gim3Δ*, *smc3-1* and *yke2Δ*, and *smc3-1* and *irc15Δ* were rather strong, as reduced colony formation of the double mutants was observed when compared to that of the respective single mutants. Interactions between *smc1-249* and *gim4Δ*, and *smc1-249* and *yke2Δ*, on the other hand, were somewhat weaker as “only” a reduction in colony size was observed for the double mutant compared to that for the respective single mutants. These new results are presented in Fig. S2 and discussed on page 7 of the revised manuscript.

4. In Figure 4C, it's clear in G2/M arrested condition that more cells in mutants (gim3, gim4, yke2 and pac10 deletion mutants) than wild type have more than one GFP spot, which suggests a defect in sister chromatid cohesion. However, it's also clear that in G1 arrested condition, more cells in these mutants than wild type have more than one spot. Is there any reasonable explanation?

There is indeed an increase of G1 cells with more than one GFP spot for the prefoldin mutants. This

© 2020. Published by The Company of Biologists under the terms of the Creative Commons Attribution License (https://creativecommons.org/licenses/by/4.0/).

may stem from chromosome mis-segregation during the previous mitosis in <10% of the cells: one daughter cell may have acquired two copies of the LacO/lacR-GFP chromosome, while the other daughter cell had none. However, the frequency of these events may be too low to become detectable by cytometry analysis, explaining why we did not detect aneuploidy as a result of chromosome mis-segeration (Fig.4 C, bottom). This point is discussed on page 8 of the revised manuscript.

5. There's no numbering in the manuscript (pdf), therefore it's not easy to point out. It is reported that cohesin has a role in DNA damage repair (Nagao K et al. Nature. 2004; McAleenan A et al. Nature. 2013). In page 6, Author described 'For example, several interactions between cohesin factors and genes involved in nucleotide excision repair, such as RAD16 and RAD1 with SMC1 and RAD10 with RAD61, in mismatch repair, such as MSH2 with MDC1 and RAD61, or in template switching, such as RAD5 with DCC1 and RMI1, might indicate a novel role for cohesin in post-replication repair' without any citation of previous reports.

The two previous reports have now been cited on page 6 of the revised manuscript.

6. In page 8. The evidences to support the subtitle 'The prefoldin complex regulates cohesion establishment' is too weak, therefore revision of this subtitle is required.

The subtitle has been changed to 'The prefoldin complex is involved in sister chromatid cohesion' in page 8 of the revised manuscript.

Second decision letter

MS ID#: JOCES/2019/237628

MS TITLE: A genetic interaction map centered on cohesin reveals auxiliary factors in sister chromatid cohesion

AUTHORS: Su Ming Sun, Amandine Batte, Mireille Tittel-Elmer, Sophie C. van der Horst, Tibor van Welsem, Gordon Bean, Trey Ideker, Fred van Leeuwen, and Haico van Attikum
ARTICLE TYPE: Research Article

I am happy to tell you that your manuscript has been accepted for publication in Journal of Cell Science, pending standard ethics checks.

Reviewer 1

Advance summary and potential significance to field

The authors used a genetic interaction screen to identify new cohesin auxiliary factors in budding yeast. They identified the microtubule-associated protein Irc15 and the prefoldin complex as new regulators of cohesin loading and cohesion. In addition, they dissect the mechanism by which these proteins affect cell-cycle activity of cohesin. These findings enhance our knowledge on cohesin regulation and its interplay with other nuclear processes. The evolutionary conservation of these factors may contribute to the understanding of pathological processes in human.

Comments for the author

The authors provide full answers to all comments and I support publication of the manuscript.

Minor reference suggestions:
1. Page 3 paragraph starting “Besides ensuring proper chromosome segregation……….and nuclear organization (Harris et al., 2014).

© 2020. Published by The Company of Biologists under the terms of the Creative Commons Attribution License (https://creativecommons.org/licenses/by/4.0/).

Add: Yamin et al, Current Genetics 2019

2. Page 3, paragraph starting “Several accessory proteins have also been implicated in promoting sister chromatid cohesion, including ……….”
Add: and Chd1 helicase (Boginya et al., Sci Rep. 2019;9(1):8929)

Reviewer 2

Advance summary and potential significance to field

Su Ming Sun et al. systematically mapped genetic interactions of 17 cohesin-related genes centered on quantitative growth measurements of >52,000 gene pairs in budding yeast. It unveiled a cohesin functional map and discovered novel involvements of microtubule-associated protein Irc15 and the prefoldin complex members in sister chromatid cohesion. The screen is comprehensive, therefore is helpful to understand cohesin interactome. The new auxiliary factors of cohesin discovered in this study provide new mechanisms regulating sister-chromatid cohesion.

Comments for the author

No further suggestion to authors, all the concerns were addressed in the revised manuscript.

© 2020. Published by The Company of Biologists under the terms of the Creative Commons Attribution License (https://creativecommons.org/licenses/by/4.0/).
